# Intraindividual comparison between open and endoscopic release in bilateral carpal tunnel syndrome: a meta‐analysis of randomized controlled trials

**DOI:** 10.1002/brb3.439

**Published:** 2016-02-16

**Authors:** Kejia Hu, Tiansong Zhang, Wendong Xu

**Affiliations:** ^1^ Department of Hand SurgeryHuashan HospitalFudan UniversityShanghaiChina; ^2^ Department of Traditional Chinese MedicineHuashan Hospital Jing‐an Branch (Jing‐an District Central Hospital)ShanghaiChina

**Keywords:** Bilateral carpal tunnel syndrome, endoscopic carpal tunnel release, intraindividual comparison, meta‐analysis, open carpal tunnel release

## Abstract

**Purpose:**

This study evaluated functional outcomes and safety after endoscopic and open bilateral carpal tunnel syndrome release in opposite hands of the same patients through a meta‐analysis of randomized controlled trial data.

**Materials and Methods:**

Randomized controlled trials involving both methods in opposite hands of patients with bilateral carpal tunnel syndrome were identified via a systematic review of PUBMED and EMBASE.

**Results:**

Relative risks (RRs) and 95% confidence intervals (CIs) from five randomized controlled trials involving 142 patients with bilateral carpal tunnel syndrome were calculated using fixed‐ or random‐effect methods, with a length of follow‐up from 24 to 52 weeks after surgery. Compared with open release, endoscopic carpal tunnel release was associated with significantly better Boston Carpal Tunnel Questionnaire functional status scores (mean difference [MD] = 0.13, 95% confidence interval [CI] [0.02 – 0.25]; *P* = 0.02), but not symptom severity scores (RR = 0.06, 95% CI [−0.15 to 0.04]; *P* = 0.25). Endoscopic release required a longer operative time, but the procedures did not differ significantly in visual analog scale pain scores (MD = 0.02, 95% CI [−0.08 to 0.11]; *P* = 0.75), handgrip strength (MD = 0.17, 95% CI [−2.03 to 2.37]; *P* = 0.88), digital sensibility static two‐point discrimination (MD = 0.34, 95% CI [−0.03 to 0.70]; *P* = 0.07), or complication rates (MD = 0.01, 95% CI [−0.02 to 0.05], *P *=* *0.47).

**Conclusion:**

From intraindividual evidence, endoscopic release promoted better recovery of daily life functions than open release, but required a longer operative time. The procedures provided similar symptom relief and hand strength and sensibility recovery, and were safe for patients with carpal tunnel syndrome.

## Introduction

Carpal tunnel syndrome (CTS) is the most frequent peripheral compressive upper limb neuropathy (Stecco and Aldegheri 2008). Conservative treatment methods, including local steroid injections, splinting, and ultrasound therapy, are often administered in mild or moderate cases, whereas complete transverse carpal ligament division is performed in cases involving severe symptoms or conservative treatment failure (Ucan et al. 2006; Scholten et al. 2007).

Traditional open carpal tunnel release (OCTR) has become the gold standard for carpal tunnel decompression since Phalen first reported this technique in the 1950s (Phalen et al. 1950). OCTR allows direct carpal tunnel visualization and guarantees complete transverse carpal ligament sectioning; however, it may lead to the formation of hypertrophic scars at the thenar, which are accompanied by pain (Aslani et al. 2012). An endoscopic carpal tunnel release (ECTR) procedure was subsequently developed, for which two techniques are commonly used: the single‐portal technique designed by Agee et al. (1992), and the two‐portal technique reported by Chow (1989) and Okutsu et al. (1989). Some surgeons favor ECTR, which is associated with reduced pain in the scar area, better esthetic results, and a more rapid return to work and daily activities (Larsen et al. 2013). In contrast, other surgeons prefer OCTR because ECTR has been associated with frequent complications and high costs and requires additional equipment (Helm and Vaziri 2003; Thoma et al. 2004).

Although some authors (Bederman et al. 2010; Bryant et al. 2006) have emphasized the importance of incorporating patients' preferences into orthopedic care, currently, decisions regarding the preferred carpal tunnel release technique are most often left to surgeons, rather than patients with CTS. Several published meta‐analyses have compared the efficacy and safety of OCTR and ECTR, but failed to address information regarding patient preferences (Chen et al. 2014; Sayegh and Strauch 2015; Zuo et al. 2015).

This meta‐analysis aimed to validate the efficacy, functional outcomes, and safety following ECTR and OCTR in opposite hands of patients with bilateral CTS. These patients are ideal candidates for determining the superiority of a particular technique from the patient's perspective, as bilateral surgery allows a direct comparison of outcomes and preferences. In particular, patients who have experienced both carpal tunnel release techniques are likely to develop a preference for one technique, based on their personal postoperative progress. To the best of our knowledge, this is the first meta‐analysis to specifically compare patients with bilateral CTS who have undergone OCTR in one hand and ECTR in the other hand, thus serving as their own internal controls.

## Materials and Methods

This meta‐analysis was performed according to the Preferred Reporting Items for Systematic Reviews and Meta‐Analyses (PRISMA) guidelines (Moher et al. 2009). As this study involved a retrospective analysis of published data, formal consent from the subjects of the various studies was not required.

### Search strategy

**Figure 1:**
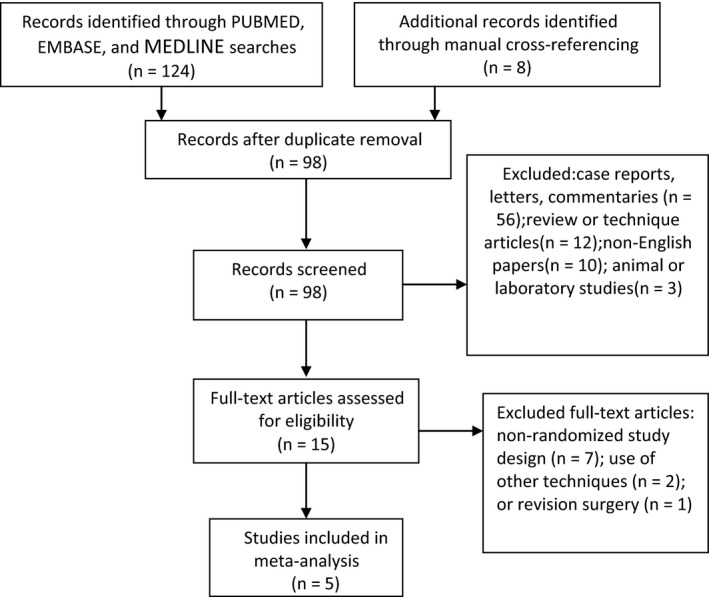
Flow diagram of the study selection process.

The PUBMED(RRID:SCR_004846), EMBASE(RRID:SCR_001650), and MEDLINE(RRID:SCR_002185) databases were searched to locate articles published between 1966 and June 2015; in addition, the Cochrane Central Register of Controlled Trials(RRID:SCR_006576) and Association Annual Congress were searched to identify articles that might have been missed in the database searches. The search strategy included various combinations of the following keywords: open, endoscopic, bilateral, CTS, carpal tunnel release, and carpal tunnel decompression. The terms “carpal tunnel release” and “carpal tunnel syndrome” were also entered into the Google search engine (RRID:SCR_008878) to identify additional trials. Only studies including bilateral carpal tunnel release were accepted for the preliminary review. Hand searches of bibliographies from published meta‐analyses and review articles were also conducted to ensure the inclusion of all pertinent studies in the preliminary review. Full manuscripts were procured and reviewed to identify eligible studies, and their citations were manually screened to identify additional studies that might have been missed. Figure 1 presents a PRISMA trial flow diagram of the study selection algorithm.

### Inclusion and exclusion criteria

We included only those RCTs for which (1) the target population included patients with a clinical diagnosis of bilateral CTS who intended to undergo bilateral carpal tunnel release; (2) an intervention that compared OCTR and ECTR with either the single‐portal or two‐portal technique; and (3) methodological criteria that included the reporting of clinically relevant results for both procedures. If the same patient cohort from a study was included in several publications, only the most recent or complete publication was selected. Studies that evaluated revision surgeries, did not report follow‐up intervals, or reported only limited qualitative findings were excluded. Case reports, letters, review, or technique articles, commentaries, non‐English papers, and animal or laboratory studies were also excluded.

### Data extraction and outcome measures

Two authors (Kejia Hu and Tiansong Zhang) assessed the methodological quality of potentially eligible studies without considering the results. Extracted data were then crosschecked between these authors to rule out any discrepancies. When necessary, authors of the previous studies were contacted to obtain missing data or additional information. For continuous outcomes with no reported standard deviations, we calculated standard deviations from the standard errors, *P* values, or confidence intervals according to the methods described in the Cochrane Handbook for Systematic Reviews of Interventions (Higgins and Thompson 2011). Outcome data and measures of variance that were reported using graphic plots, but omitted from the body of the text were quantified using plot‐digitizing software (Plot Digitizer Version 2.6.4; Joseph Huwaldt and Scott Steinhorst, http://www.plot-digitizer.com-about.com/).

The following data were extracted independently for each study: sample size, follow‐up duration, symptom duration, diagnostic electrophysiologic testing, and surgical technique. The primary outcomes were the recovery of function and relief of symptoms, which assessed according to the symptom severity and functional status components of the validated Boston Carpal Tunnel Questionnaire (BCTQ‐S and ‐F, respectively) (Levine et al. 1993). Secondary outcomes included maximum handgrip strength and static two‐point discrimination (2PD), interval between the procedure and return to work, and operative times of the two procedures were analyzed and compared.

### Statistical analysis

The statistical analysis was conducted using Review Manager (RevMan) software, version 5.1 (The Nordic Cochrane Centre, Cochrane Collaboration, Copenhagen, Denmark) (RRID:SCR_003581). Continuous data were analyzed via the inverse‐variance statistical method and computations of the standardized mean difference (SMD) or mean difference (MD) and 95% confidence interval (CI). Dichotomous data were analyzed using the Mantel–Haenszel statistical method and computations of the risk ratio (RR) and 95% CI. *I* ^2^ statistics was used to evaluate between‐study heterogeneity in this meta‐analysis (Higgins and Thompson 2002; Higgins et al. 2003; Patsopoulos et al. 2008). The random‐effects model was used when obvious heterogeneity was observed among the included studies (*I* ^2^ > 50%). The fixed‐effects model was used when no significant heterogeneity was observed between the included studies (*I* ^2^ ≤ 50%). Begg's funnel plot and Egger's test for possible publication bias were not used, as fewer than 10 studies were included in each risk factor category.

## Results

### Eligible studies

Five RCTs (Ferdinand and MacLean 2002; Wong et al. 2003; Rab et al. 2006; Kang et al. 2013; Michelotti et al. 2014) published between 2002 and 2014 were included in this meta‐analysis (Table 1). These studies were of moderate or high quality (NOS scores >5), and included a combined total number of 142 patients at the time of the final follow‐up. Each trial was designed to compare OCTR and ECTR in patients with bilateral CTS. The cohorts of the included RCTs ranged from 10 to 52 patients, with follow‐up durations ranging from 24 to 52 weeks. The main characteristics of the included studies are summarized in Table 1.

**Table 1:** Characteristics of studies included in the meta‐analysis

Author group (Year)	Country	Study type	Intervention	Sample size (ECTR/OCTR)	Length of Follow‐up (weeks)	Outcomes for analysis
Ferdinand and MacLean (2002)	UK	RCT	ECTR vs. OCTR	(25/25)	52	Operative time, satisfaction rating, grip strength, Jebsen score, static 2PD, complications
Wong et al. (2003)	China	RCT	ECTR vs. OCTR	(30/30)	52	Operative time, VAS (pain), ADL score, symptom relief, grip strength, pinch strength, distal latency, NCV, complications
Rab et al. (2006)	Austria	RCT	ECTR versus OCTR	(10/10)	52	VAS (pain), BCTQ‐S, BCTQ‐F, grip strength, pinch strength, key pinch strength, static 2PD, distal latency, NCV, complications
Kang et al. (2013)	Korea	RCT	ECTR versus OCTR	(52/52)	36	Treatment preference, BCTQ‐S, BCTQ‐F, DASH, complications, reoperation
Michelotti et al. (2014)	USA	RCT	ECTR versus OCTR	(25/25)	24	VAS (pain), BCTQ‐S, BCTQ‐F, static 2‐PD, SW, thenar strength, grip strength, satisfaction, complications

ECTR, endoscopic carpal tunnel release; OCTR, open carpal tunnel release; RCT, randomized controlled trial; 2PD, two‐point discrimination; VAS, visual analog scale; ADL, activities of daily living; NCV, nerve conduction velocity; BCTQ‐S, Boston Carpal Tunnel Questionnaire symptom severity score; BCTQ‐F, BCTQ functional status score; DASH, Disabilities of the Arm, Shoulder, and Hand; SW, Semmes–Weinstein monofilament testing.

### Meta‐analysis

#### Functional outcomes

**Figure 2:**
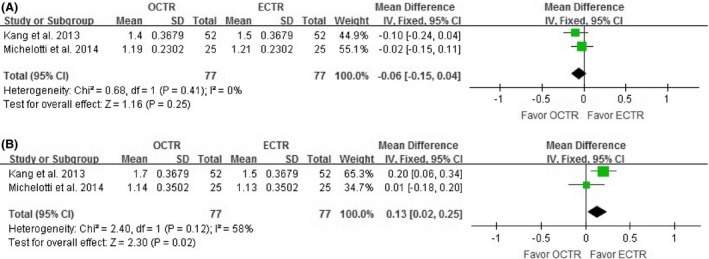
Forest plot of the Boston Carpal Tunnel Questionnaire Symptom Severity scores (BCTQ‐S) (A) and Functional Status scores (BCTQ‐F) (B). OCTR, open carpal tunnel release; ECTR, endoscopic carpal tunnel release; SD, standard deviation; IV, inverse‐variance; CI, confidence interval.

Two RCTs (Kang et al. 2013; Michelotti et al. 2014), including 77 bilateral hands, reported the study patients' BCTQ scores; compared with OCTR, ECTR was not associated with a significantly increase in BCTQ‐S scores (MD = 0.06, 95% CI [−0.15 to 0.04]; *n* = 77; *I* ^2^ = 0%, *P* = 0.25; Fig. 2A), but did yield a significant improvement in BCTQ‐F scores (MD = 0.13, 95% CI [0.02–0.25]; *n* = 77; *I* ^2^ = 58%, *P* = 0.02; Fig. 2B).

#### VAS pain scores

**Figure 3:**
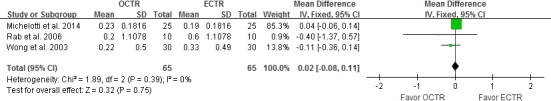
Forest plot of a comparison of visual analog scale pain scores. OCTR, open carpal tunnel release; ECTR, endoscopic carpal tunnel release; SD, standard deviation; IV, inverse‐variance; CI, confidence interval.

Pooled data from three trials (Wong et al. 2003; Rab et al. 2006; Michelotti et al. 2014), including 65 bilateral hands, indicated that there was no statistical difference between ECTR and OCTR in terms of VAS pain score improvement (MD = 0.02, 95% CI [−0.08 to 0.11]; *n* = 65; *I* ^2^ = 0%, *P* = 0.75; Fig. 3).

#### Operative duration

**Figure 4:**
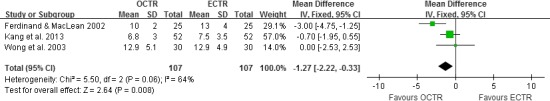
Forest plot of a detailed comparison of operative durations. OCTR, open carpal tunnel release; ECTR, endoscopic carpal tunnel release; SD, standard deviation; IV, inverse‐variance; CI, confidence interval.

Three studies (Ferdinand and MacLean 2002; Wong et al. 2003; Kang et al. 2013) evaluated differences in operative durations between the ECTR and OCTR groups. Most researchers (Okutsu et al. 1989; Ozyürekoğlu et al. 2006; Patsopoulos et al. 2008; Larsen et al. 2013) reported that ECTR surgery required a longer operative time. Pooled data from three studies indicated a significantly reduced operative time with OCTR, compared to ECTR (MD = −1.27, 95% CI [−2.22 to −0.33], test for overall effect: *Z *=* *2.64; *P *=* *0.008; Fig. 4).

#### Handgrip strength

**Figure 5:**
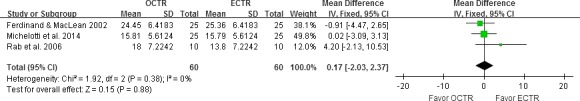
Comparison of handgrip function recovery at least 24 weeks after surgery. OCTR, open carpal tunnel release; ECTR, endoscopic carpal tunnel release; SD, standard deviation; IV, inverse‐variance; CI, confidence interval.

Data were pooled from three trials (Ferdinand and MacLean 2002; Rab et al. 2006; Michelotti et al. 2014), including 60 hands, to compare handgrip strength at an interval of at least 24 weeks after surgery. The result suggested a lack of statistical difference between ECTR and OCTR in terms of handgrip strength recovery (MD = 0.17 kg, 95% CI [−2.03 to 2.37]; *n* = 60; *I* ^2^ = 0%, *P* = 0.88; Fig. 5), indicating that the outcome did not favor a particular carpal tunnel release technique at a long‐term follow‐up.

#### Digital sensibility static 2PD

**Figure 6:**
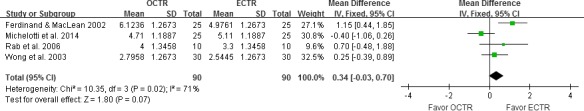
Comparison of postoperative two‐point discrimination (2PD) recovery. OCTR, open carpal tunnel release; ECTR, endoscopic carpal tunnel release; SD, standard deviation; IV, inverse‐variance; CI, confidence interval.

An analysis of pooled data from four trials (Ferdinand and MacLean 2002; Wong et al. 2003; Rab et al. 2006; Michelotti et al. 2014), including 90 hands, revealed a lack of significant difference between ECTR and OCTR in terms of digital sensibility static 2PD recovery (MD = 0.34 kg, 95% CI [−0.03 to 0.70]; *n* = 60; *I* ^2^ = 71%, *P* = 0.07; Fig. 6).

#### Complication rates

**Figure 7:**
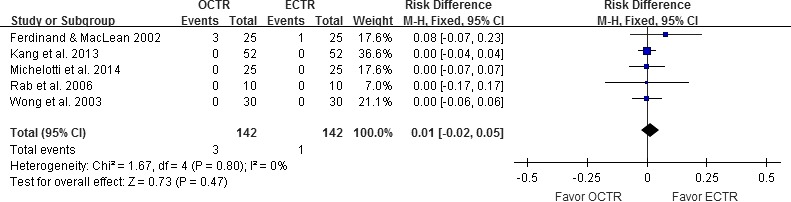
Forest plot of the overall complication rate analysis. OCTR, open carpal tunnel release; ECTR, endoscopic carpal tunnel release; SD, standard deviation; M‐H, Mantel‐Haenszel; CI, confidence interval.

All five studies included complete complication rate data and were thus included in the analysis of overall complication rates (Ferdinand and MacLean 2002; Wong et al. 2003; Rab et al. 2006; Kang et al. 2013; Michelotti et al. 2014). Complications included irreversible nerve damage, reversible nerve problems (including neurapraxia and numbness), wound problems, and reflex sympathetic dystrophy. Subgroup comparison was not performed because of the small amount. A pooled data analysis found that the complication rates for ECTR and OCTR did not significantly differ (RR = 0.01, 95% CI [−0.02, 0.05], test for overall effect: *Z *=* *0.73, *P *=* *0.47; Fig. 7).

### Publication bias

A funnel plot and Egger's test were used to evaluate publication bias in the included literature. The funnel plot shape did not reveal any obvious asymmetry (data not shown), indicating a lack of bias.

## Discussion

Generally, surgical transverse carpal ligament release yields satisfactory results, regardless of technique (Trumble et al. 2002). Although the standard OCTR technique is known to be effective and safe, ECTR was developed to reduce the incision size and time required for recovery (Brown et al. 1993). Despite considerable efforts to determine a superior technique, discussion remains regarding whether the benefits of reduced daily life impairments associated with endoscopic carpal tunnel surgery outweigh the drawbacks of relative technical difficulty, cost‐effectiveness, time requirements, and potential risks of iatrogenic injury to neurovascular structures (Jacobsen and Rahme 1996; Chung et al. 1998; Bhattacharya et al. 2004; Cresswell et al. 2008; Atroshi et al. 2009).

Our meta‐analysis included data from five randomized controlled trials involving a total of 142 patients with bilateral CTS. All patients underwent OCTR on one hand and ECTR on the opposite hand and thus served as their own internal controls (Ferdinand and MacLean 2002; Wong et al. 2003; Rab et al. 2006; Kang et al. 2013; Michelotti et al. 2014). This significant strength of our study reduced bias associated with the reporting of subjective patient variables, particularly pain and self‐perception. Additionally, although many study outcomes include patients' reports of symptom relief and functional improvement, these subjective results are not standardized or properly measured (Chen et al. 2014; Sayegh and Strauch 2015; Zuo et al. 2015).

The self‐applied BCTQ was developed to scientifically evaluate subjective results following surgical intervention and is reproducible, coherent, valid, and sensitive to clinical changes. BCTQ‐S evaluates the severity, frequency, duration, and type of symptoms, whereas BCTQ‐F evaluates the effects of CTS on daily life (Leite et al. 2006). In a recent systematic meta‐analysis by Sayegh and Strauch (2015), patients who underwent ECTR could return to work sooner according to the validated BCTQ‐S and BCTQ‐F indices. In our study, which involved opposite hands of the same patients, the evidence indicated that ECTR and OCTR both reduced symptom severity, compared with the preoperative state. ECTR was found to provide significant improvements in hand functional recovery, which might allow patients to achieve larger gains in daily living and self‐care activities, such as personal care, work, housekeeping, and leisure, within a shorter postoperative time interval. However, we should figure that a minor change of BCTQ‐f may not have an impact to the patient when we consider the minimal clinically important difference (MCID), although it has statistical significant. Leite et al. (2006) noted that when the MCID is 0.74 for the BCTQ (total score based on the average of both subscales with scale ranges from 1 to 5, a value considered superior to generic measures, in distinguishing clinically important differences after carpal tunnel release. Ozyürekoğlu et al. (2006) also indicated that a decrease of 1.04 or more in the Symptom Severity Scale (SSS) of the CTS Questionnaire may indicate a clinically important change in a patient's state of health; this amount of reduction in the SSS score is quite large considering that the range of scores is from 0 to 5.0. So, we think further research is needed and also maybe examine the consistency of BCTQ's properties, using appropriate statistical measures and defining the MCID for each subscale against appropriate external criteria.

In addition, our meta‐analysis of long‐term follow‐up results established equal achievements in handgrip strength recovery at least 24 weeks after undergoing either surgery. Thoma et al. (2004) analyzed data pooled from three studies and found no significant difference in the time required to return to work. However, Chen et al. (2014), who included additional RCTs not included in the Thoma study, found that ECTR reduced the time required to return to work by 8 days, compared with OCTR. In contrast, our meta‐analysis did not compare return‐to‐work data because the included studies involved bilateral hand procedures; furthermore, we believe this information was difficult to determine because the time at which a patient returns to work is often indicated by the surgeon, regardless of the procedure type.

Our meta‐analysis revealed that, compared with OCTR, ECTR required a longer operative time; however, other meta‐analyses reported different conclusions. For example, Sayegh and Strauch (2015) indicated a shorter operative time for endoscopic versus open procedures, whereas Zuo et al. (2015) reported no difference in the operative times associated with both procedures. Notably, the learning curve for each method must be considered when evaluating the results and outcomes, and proper training and experience in both techniques is required to achieve proficiency (Malaviya 2008).

Our comparison of opposite hands in the same patients indicated a lack of statistical differences between the two procedures in several parameters. First, relief of pain and paresthesia are important criteria for evaluating the effectiveness of these surgical methods. The Thoma et al. (2004) and Chen et al. (2014) studies both reported a lack of statistical differences in short‐term or long‐term pain outcomes between patients who underwent ECTR and OCTR. In our meta‐analysis, in which the VAS was used to quantify patients' complaints of pain and minimize subjective influences, we also did not observe statistical differences between ECTR and OCTR. Second, our meta‐analysis did not find statistical differences in digital sensibility static 2PD recovery between the two procedures, similar to the results reported by Sayegh and Strauch (2015). Third, complication rates can be used to evaluate methodological safety. Previously, Helm and Vaziri (2003) reported a higher rate of reversible nerve problems and lower rate of wound complications among ECTR‐treated hands; these authors believed that most reversible nerve problems would resolve within a few weeks and were probably related to neuropraxia due to the instrumentation. Only one study (Ferdinand and MacLean 2002) in our analysis, however, reported complications, but found no statistical difference between ECTR and OCTR. We therefore consider both ECTR and OCTR to be safe for patients with CTS.

However, we must carefully consider some possible limitations of our study when interpreting the results. First, although we included only RCTs, all of the trials had methodological flaws, including a failure to blind the outcome assessor or lack of a baseline outcome evaluation. Second, two ECTR operative techniques (single‐portal and two‐portal) were used in the trials, and these techniques might be associated with different outcomes (Chow 1989; Agee et al. 1992; Atroshi et al. 2009; Kang et al. 2013). However, we were unable to perform a subgroup analysis because of the rather small number of studies. Finally, we only included English language studies. Despite our best efforts, which included multiple search methods, we might have omitted non‐English language trials that would have been applicable to our meta‐analysis.

Previously, OCTR was considered the gold standard carpal tunnel decompression procedure (Phalen et al. 1950); however, our intraindividual comparison analysis of patients with bilateral CTS who underwent OCTR in one hand and ECTR in another suggested that both procedures were tolerated well, with no differences in hand strength or sensibility recovery. Although ECTR was associated with a better recovery of daily life functions, it required a longer operative time. Ultimately, both ECTR and OCTR were found to be safe for patients with CTS. Accordingly, we recognize that as surgical proficiency in endoscopic surgery increases, ECTR will be more widely used to treat CTS in the future.

## Conflict of Interest

The authors have no conflicts of interest to declare.
